# Decreased Helios Expression in Regulatory T Cells in Acute Coronary Syndrome

**DOI:** 10.1155/2017/7909407

**Published:** 2017-11-12

**Authors:** Lili Jiang, Feng Chen, Xiaofan Hu, Yingying Hu, Yange Wang, Wenyong Zhang, Yudong Peng, Longxian Cheng

**Affiliations:** ^1^Laboratory of Cardiovascular Immunology, Institute of Cardiology, Union Hospital, Tongji Medical College, Huazhong University of Science and Technology, Wuhan 430022, China; ^2^Department of Geriatrics, Li Yuan Hospital, Tongji Medical College, Huazhong University of Science and Technology, 39 Yanhu Avenue, Wuhan 430077, China

## Abstract

Regulatory T cells (Tregs) play an essential role in acute coronary syndrome (ACS). However, there is debate about which Treg subsets are truly critical to ACS. Helios, a transcription factor, was recently reported to be a bona fide marker for natural Tregs or activated Tregs with a suppression function, but little is known about its role in ACS. We therefore examined Helios+ Tregs in patients with ACS, patients with stable angina, and control subjects. 73 patients with ACS, 30 patients with stable angina, and 48 control subjects were enrolled. The frequencies and estimated absolute numbers of different Treg subsets in peripheral blood were measured by flow cytometry. Plasma cytokine level was measured by ELISA. The mRNA expression of Foxp3 and Helios in purified CD4+ T cells was determined by RT-PCR. Helios+ Tregs was decreased significantly in patients with ACS. The frequency and estimated absolute numbers of CD4+Foxp3+Helios+ Tregs were negatively correlated with IL-6 and positively correlated with circulating level of TGF-beta1 and HDL-C. The mRNA expression of Foxp3 and Helios was decreased in CD4+ T cells from patients with ACS. In summary, Helios+ Tregs was downregulated in patients with ACS and may play a role in ACS.

## 1. Introduction

Coronary artery disease (CAD) is a leading cause of death worldwide [1]. Immunological inflammatory responses play a pivotal role in its progression. A series of immune cells such as macrophages and monocytes and different subsets of lymphocytes participate in the chronic inflammatory response and eventually initiate the progression to acute coronary syndrome [2–4].

Regulatory T cells (Tregs)—an important subset of the lymphocyte population—are capable of suppressing pathogenic T cells and inflammatory responses [5], to maintain immune system homeostasis. It has been revealed that an abnormal quantity or dysfunction of Tregs might be associated with many different conditions, including carcinoma [6], diabetes [7], organ transplant reactions [8], systemic autoimmune disorders [9], and CAD [10–12]. A few studies have shown that a downregulation in Tregs might contribute to the development of ACS [11, 12], although others have reported conflicting results, some describing an upregulation of Tregs in patients with ACS [13–15], and others suggesting no significant finding in patients with ACS [16, 17]. Meanwhile, the definition of Treg marker patterns has long been controversial. It has been regarded as the traditional CD4+CD25+ T cell pattern discovered 30 years ago [18] or the CD4+CD25+Foxp3+ T cell pattern found later [19]. The nuclear transcriptional factor Foxp3 was once considered a canonical marker for Tregs. However, researchers have found that Foxp3 expression may occur in Tregs with a suppressive function, as well as cytokine-producing effector T cells without a suppressive function [20–22]. Similarly, CD25 may be transiently upregulated in newly activated conventional CD4+ T cells [23, 24]. In view of the variable expression patterns and the instability of these markers, it is difficult to determine which markers are reliable.

Given the conflicting opinions on Tregs in ACS and the variable and unstable markers mentioned above, more stable and reliable markers are still required to better distinguish patients with ACS from others and to identify regulatory cells. The zinc finger transcription factor Helios, a member of the Ikaros family, was thought to be specifically expressed in thymus-derived CD4+Foxp3+ nTregs in both mice and humans [25, 26]. In addition, Helios has been acknowledged to be a mediator in T lymphocyte immune homeostasis [27] and a marker linked to T cell immune tolerance [28, 29]. Animal studies have shown that Helios is required for stable inhibitory activity of CD4+Foxp3+ Tregs [30], whereas some other reports suggest that, rather than being a marker of nTregs, Helios could be a good marker for activated Tregs with a suppressive function [28, 31]. Foxp3+Helios+ T cells have been reported to play a vital regulatory role in immunological balance [32, 33]. It has been discovered that the coexpression of Foxp3 and Helios represents an important functional state of Tregs [34]. To the best of our knowledge, no research on Helios+ Tregs in CAD has been reported.

Cytokines play a critical role in immunological regulation. Transforming growth factor beta1 (TGF-beta1) was reported to be a key cytokine for the peripheral induction of regulatory T cells [35]. IL-6 was demonstrated to attenuate the development of Helios+ Tregs induced by TGF-beta [36]. Both of these cytokines play essential roles in ACS. The alteration of such cytokines and their relationship with Helios+ Tregs in ACS are in need of further clarification.

In this study, we measured the expression levels of CD25, Foxp3, and Helios in the peripheral blood of patients with ACS and explored their possible significance in CAD. The levels of Treg-associated cytokines were detected to investigate their potential relation with Tregs in ACS.

## 2. Materials and Methods

### 2.1. Ethics

The investigation strictly conformed to the principles in the Declaration of Helsinki. Our research was approved by the ethics committee of Tongji Medical College, Huazhong University of Science and Technology. All volunteers gave informed consent.

### 2.2. Subjects

This trial followed a cohort of 151 subjects recruited from the Union Hospital, Tongji Medical College, Huazhong University of Science and Technology (Wuhan, China). The patients were divided into three groups: (1) a control group (*n* = 48, 27 men and 21 women, 54.73 ± 1.5 years of age) consisting of patients without coronary heart disease history or abnormal coronary arteries on angiography; (2) a stable angina (SA) group (*n* = 30, 18 men and 12 women, 57.50 ± 1.5 years of age) with patients who showed typical exertional chest discomfort accompanied by down-sloping or horizontal ST segment depression N1 mm in an exercise test; and (3) an ACS group (*n* = 73, 55 men and 18 women, 58.32 ± 1.0 years of age), which included patients who presented with chest pain at rest with ischemic electrocardiographic alterations, including ST segment alterations and T-wave inversions, or a remarkable increase in serum levels of creatine kinase MB (≥6.6 ng/ml) and troponin I (≥262 pg/ml). The baseline characteristics of the subjects in all three groups are presented in Table 1. Subjects were excluded if they had been treated with anti-inflammatory medication or had a history of renal deficiency, advanced liver disease, malignant conditions, thromboembolism, collagen disease, or other inflammatory diseases.

### 2.3. Preparation of Blood Samples and Isolation of Peripheral Blood Mononuclear Cells

After an 8-hour fast, on the morning of the admission day, blood samples were drawn with a 21-gauge needle for spotless antecubital venipuncture and collected into sodium heparin-coated tubes. The blood samples were centrifuged at 300*g* for 7 min, and the plasma was aliquoted and then stored at −80°C for further use. Peripheral blood mononuclear cells in the blood were isolated with Ficoll-Hypaque (Sigma, USA) by density gradient centrifugation and then washed twice before flow cytometry.

### 2.4. Isolation of CD4+ T Cells

CD4+ T cells were isolated using CD4 microbeads (Miltenyi Biotec, Germany) according to the manufacturer's instructions. The purity of CD4+ T cells was >90% as assessed by fluorescence-activated cell sorting. These CD4+ T cells were then used for real-time polymerase chain reaction (RT-PCR).

### 2.5. Flow Cytometric Analysis

Peripheral blood mononuclear cells were harvested and stained with the following antibodies according to the manufacturer's protocols: PerCP-conjugated anti-human CD4 (clone: 61D3, BioLegend), PE-CY7-conjugated anti-human CD25 (clone: M-A251, Biosciences, USA), APC-conjugated anti-human Foxp3 (clone: PCH101, eBioscience), and PE-conjugated anti-Helios (clone: 22F6, eBioscience). Corresponding isotype antibodies were used. According to the manufacturer's instructions, fixation and permeabilization were carried out after the staining of cell surface antigens CD4 and CD25, for the subsequent intracellular staining of FOXP3 and Helios. The cells were tested by flow cytometry with a fluorescence-activated cell sorter (BD Biosciences, USA). The results were analyzed with FlowJo v.X.0.7 (Treestar Inc., USA). The calculation formula [37] for the estimated absolute numbers of different cell subsets was listed in Table 1.

### 2.6. RT-PCR

Total RNA of the CD4+ T cells was extracted using Trizol reagent (Takara, Japan) according to the manufacturer's instructions. cDNA was obtained with a reverse transcriptase kit (Takara, Japan). The expression levels of genes were detected by SYBR Green nucleic acid gel stains (Takara, Japan) with the −2^ΔΔCT^ method; finally, all samples were analyzed with an ABI Prism 7900 Sequence Detection System (Applied Biosystems, USA). Primer sequences used were as follows: human GAPDH (F: 5′-CCACATCGCTCAGACACCAT-3′, R 5′-GGCAACAATATCCACTTTACCAGAGT-3′), human Foxp3 (F: 5′-GGCTGGTCTGCTTGAGAAAC-3′, R: 5′-ATTGCCAAACTGTGGTC TCC-3′), and human Helios (F: 5′-CTTTCCAAGACACACTTCACCA-3′, R: 5′-TA TCTCCTTTGTTACCGCTTCC-3′). All samples were amplified for 40 cycles in duplicate, and the expression levels of these genes were normalized to the levels of GAPDH. A sample from a healthy volunteer was used as the control sample against which all measures were compared [12, 37].

### 2.7. Enzyme-Linked Immunosorbent Assay for the Detection of Patients' IL-6 and TGF-Beta1

Plasma samples from the subjects were collected and stored at −80°C until the cytokine levels were tested with specific enzyme-linked immunosorbent assay kits (Neobioscience, China) according to the manufacturer's instructions. Each assay was carried out in triplicate.

### 2.8. Statistical Analysis

The data are shown as the mean ± SEM or as a percentage. Significant differences among the three groups of patients were tested by one-way analysis of variance and Pearson chi-square tests. Significant differences between two groups were examined by independent-samples *t*-tests. Spearman's correlation analysis was used to determine the correlation between the variables. A *P* value of less than 0.05 was considered to indicate statistical significance. SPSS 20.0.0 was used for the statistical analyses.

## 3. Results

### 3.1. Baseline Characteristics of the Subjects

The clinical data of all enrolled subjects are listed in Table 2. There were no remarkable differences between the groups with respect to gender, age, smoking, diabetes mellitus, hypertension, or hyperlipidemia (*P* > 0.05). No significant difference was found in systolic or diastolic blood pressure or in serum levels of total cholesterol, low-density lipoprotein cholesterol, or triglycerides (*P* > 0.05). The serum level of high-density lipoprotein cholesterol (HDL-C) was significantly decreased in the ACS group compared with the SA and control groups (*P* < 0.05). There was a significant difference in statin use among the three groups and we compared the CD4+Fxop3+Helios+ levels between subjects with and without statin use, and no significant difference was found. The serum levels of creatinine kinase MB and troponin I were remarkably increased in the ACS group compared with the SA and control groups (*P* < 0.001).

### 3.2. CD4+Foxp3+Helios+ T Cells in ACS Patients Was Significantly Decreased

**Figure 1:**
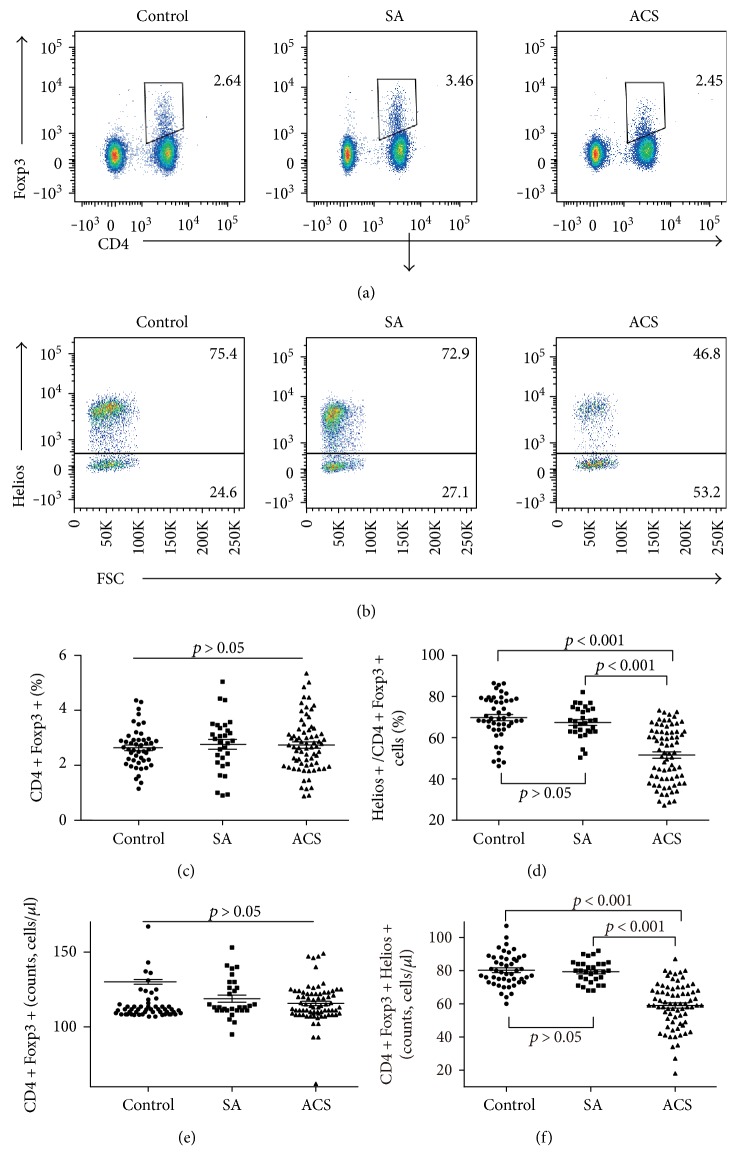
The Helios+ Tregs is significantly decreased in patients with ACS. (a) Representative images of the frequency of CD4+Foxp3+ T cells from a single patient in each group. (b) Representative images of the proportion of Helios+ cells in CD4+Foxp3+ T cells from a single patient in each group. (c) The percentage of CD4+Foxp3+ T cells among lymphocytes for each group. (d) The percentage of CD4+Foxp3+Helios+ T cells among CD4+Foxp3+ T cells for each group. (e) The estimated absolute number of CD4+Foxp3+ T cells for each group. (f) The estimated absolute number of CD4+Foxp3+Helios+ T cells for each group.

The proportion of Helios+ cells in CD4+Foxp3+ T cells was significantly lower in patients with ACS (52.26% ± 1.54%, *P* < 0.001) than in patients in the SA group (67.36% ± 1.38%) and the control group (69.75% ± 1.51%) (Figures 1(b) and 1(d)). However, the percentage of CD4+Foxp3+ T cells (Figures 1(a) and 1(c)) showed no significant difference among the three groups. The estimate absolute numbers of CD4+Foxp3+Helios+ T cells were significantly lower in patients with ACS (59.0% ± 1.6%, *P* < 0.001) than in patients in the SA group (79.3% ± 1.2%) and the control group (80.2% ± 1.4%) (Figure 1(f)). The estimate absolute numbers of CD4+Foxp3+ T cells showed no significant difference among the three groups (Figure 1(e)).

### 3.3. CD4+CD25+, CD4+CD25+Foxp3+, and CD4+CD25+Foxp3+Helios+ T Cells in the Peripheral Blood of ACS Patients

**Figure 2:**
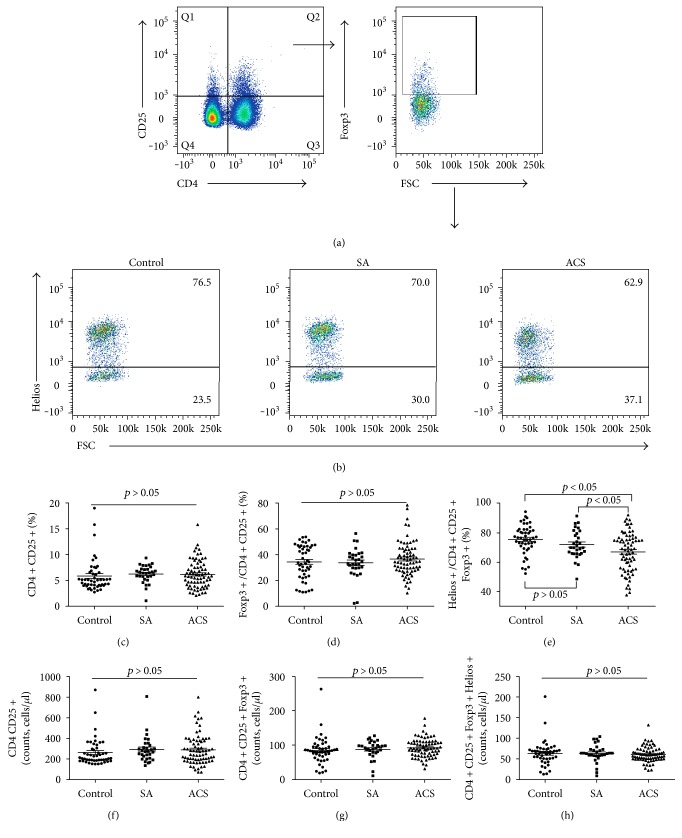
CD25 does not contribute to better distinguishing ACS patients from others. (a) CD4+CD25+ and CD4+CD25+Foxp3+ T cells were gated by flow cytometry. (b) Representative images of the proportion of Helios+ cells in CD4+CD25+Foxp3+T cells from a single patient in each group. (c) The percentage of CD4+CD25+ T cells among lymphocytes for each group. (d) The percentage of CD4+CD25+Foxp3+ T cells among CD4+CD25+ T cells for each group. (e) The percentage of CD4+CD25+Foxp3+Helios+ T cells among CD4+CD25+Foxp3+ T cells for each group. (f) The estimated absolute number of CD4+CD25+ T cells for each group. (g) The estimated absolute number of CD4+CD25+Foxp3+ T cells for each group. (h) The estimated absolute number of CD4+CD25+Foxp3+Helios+ T cells for each group.

No significant difference was found in the frequencies of CD4+CD25+ T cells (Figure 2(c)) or CD4+CD25+Foxp3+ T cells (Figure 2(d)) among the three groups. Only one significant difference was found in the expression of Helios in CD4+CD25+Foxp3+ T cells, which was lower in patients with ACS (67.10% ± 1.55%, *P* < 0.01) than in the SA group (72.07% ± 1.74%) and the control group (75.66% ± 1.41%) (Figures 2(b) and 2(e)). The estimated absolute numbers of CD4+CD25+ T cells, CD4+CD25+Foxp3+ T cells, and CD4+CD25+Foxp3+Helios+ T cells showed no significant difference among the three groups (Figures 2(f), 2(g), and 2(h)).

### 3.4. The mRNA Expression of Both Foxp3 and Helios Decreased in the CD4+ T Cells of Patients with ACS

**Figure 3:**
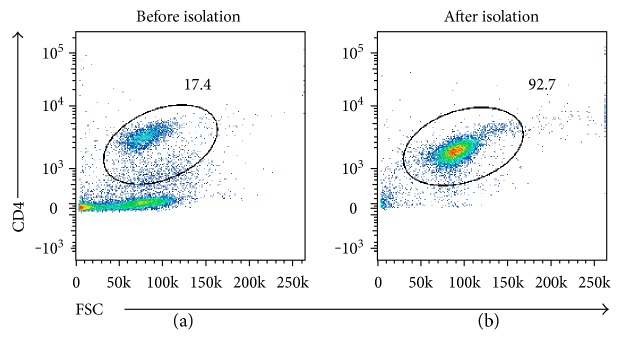
Purity of the CD4+ cells isolated using magnetic selection. (a) Dot plot showing the purity of the CD4+ cells before isolation. (b) Dot plot showing the purity of the CD4+ cells after isolation.

**Figure 4:**
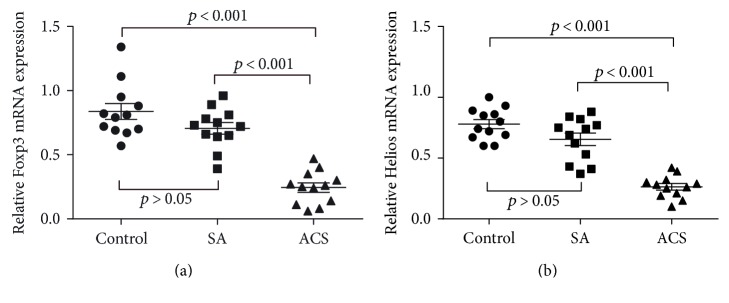
Both Foxp3 and Helios mRNA expression were remarkably decreased in CD4+ cells from ACS patients compared with patients in the SA and control groups. RT-PCR was used to detect the mRNA expression of Foxp3 (a) and Helios (b).

The mRNA expression of Foxp3 and Helios in CD4+ T cells (Figure 3) was quantified by RT-PCR. The ACS patients were characterized by significantly lower mRNA expression of both Foxp3 and Helios (Figure 4). Although flow cytometry detected no significant differences in the expression of Foxp3+ Tregs among the three groups, a significant decrease in its mRNA expression did exist. In contrast, the expressions of Helios in both testing methods were comparable. Helios exhibited better consistency in mRNA and protein level than Foxp3 did.

### 3.5. The Correlation of Circulating Cytokines and HDL-C with Helios+ Tregs

**Figure 5:**
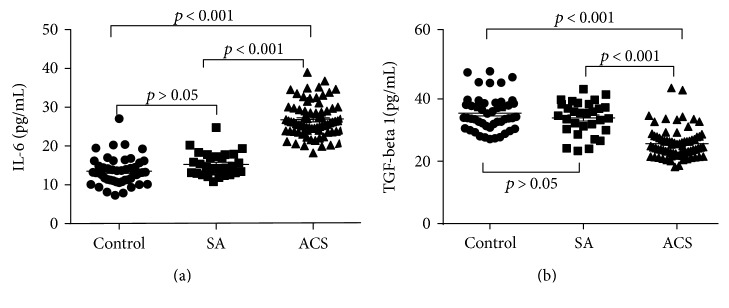
Levels of plasma cytokines in ACS patients. The plasma concentrations of IL-6 (a) and TGF-beta1 (b) were determined by ELISA.

**Figure 6:**
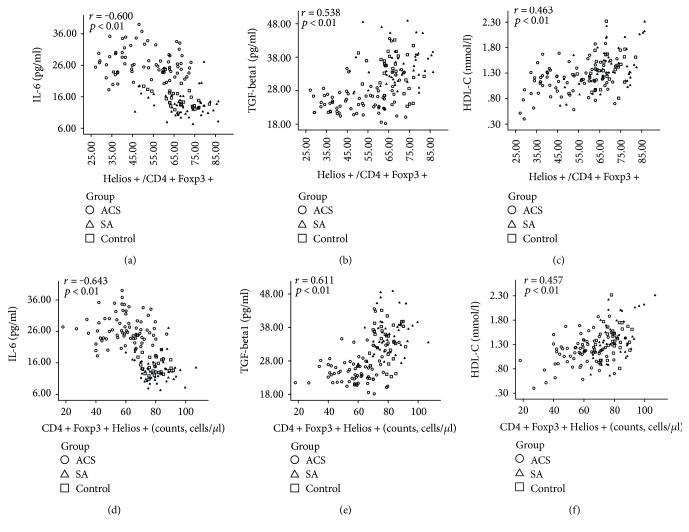
Spearman correlation analysis of the frequency of Helios+ Tregs with circulating cytokines and HDL-C in patients in the control, SA, and ACS groups. (a) The plasma level of IL-6 was negatively correlated with the proportion of Helios+ cells in CD4+Foxp3+ T cells (*r* = −0.600, *P* < 0.01). (b) The plasma level of TGF-beta1 was positively correlated with the proportion of Helios+ cells in CD4+Foxp3+ T cells (*r* = 0.538, *P* < 0.01). (c) The serum level of HDL-C was positively correlated with the proportion of Helios+ cells in CD4+Foxp3+ T cells (*r* = 0.565, *P* < 0.01). (d) The plasma level of IL-6 was negatively correlated with the CD4+Foxp3+Helios+ cell counts (*r* = −0.643, *P* < 0.01). (e) The plasma level of TGF-beta1 was positively correlated with the CD4+Foxp3+Helios+ cell counts (*r* = 0.611, *P* < 0.01). (f) The plasma level of HDL-C was positively correlated with the CD4+Foxp3+Helios+ cell counts (*r* = 0.457, *P* < 0.01).

Cytokines are pivotal players in immune responses during atherosclerosis progression. To determine the relationship between cytokines and Helios+ Tregs, the plasma levels of IL-6 and TGF-beta1 were examined. The concentration of IL-6 was significantly higher in the ACS group (26.75 ± 0.52 pg/ml) than in the SA group (15.28 ± 0.55 pg/ml) and the control group (13.53 ± 0.53 pg/ml) (*P* < 0.001) (Figure 5(a)). The concentration of TGF-beta1 was significantly lower in the ACS group (25.69 ± 0.56 pg/ml) than in the SA group (33.90 ± 0.97 pg/ml) and the control group (35.40 ± 0.82 pg/ml) (*P* < 0.001) (Figure 5(b)). Next, we performed a correlation analysis of Helios+ Tregs with the circulating cytokines and HDL-C in all three groups. The plasma level of IL-6 was negatively correlated with the proportion of Helios+ cells in CD4+Foxp3+ T cell (*r* = −0.600, *P* < 0.01) (Figure 6(a)). The plasma level of TGF-beta1 was positively correlated with the proportion of Helios+ cells in CD4+Foxp3+ T cells (*r* = 0.538, *P* < 0.01) (Figure 6(b)). The correlations between the proportion of Helios+ cells in CD4+Foxp3+ T cell and IL-6 or TGF-beta1 seemed to be partly driven by the difference between the groups (Table 3). Such a situation may be partly due to the limited sample size in each group. The serum level of HDL-C was positively correlated with the proportion of Helios+ cells in CD4+Foxp3+ T cells (*r* = 0.565, *P* < 0.01) (Figure 6(c)). The plasma level of IL-6 was negatively correlated with the CD4+Foxp3+Helios+ cell counts (*r* = −0.643, *P* < 0.01) (Figure 6(d)). The plasma level of TGF-beta1 was positively correlated with the CD4+Foxp3+Helios+ cell counts (*r* = 0.611, *P* < 0.01) (Figure 6(e)). The plasma level of HDL-C was positively correlated with the CD4+Foxp3+Helios+ cell counts (*r* = 0.457, *P* < 0.01) (Figure 6(f)).

## 4. Discussion

Here, we show for the first time that Helios+ Tregs are significantly decreased in ACS. Furthermore, the expression level of Helios is negatively correlated with circulating IL-6 but positively correlated with TGF-beta1 and HDL-C.

Tregs are an important type of T cell that can suppress inflammatory responses and reduce plaque formation and have a potential role in the attenuation of atherosclerosis initiation and evolution. Although several studies have investigated the role of Tregs in ACS, the changes in Treg levels and the expression patterns of Tregs in ACS patients remain controversial. Some studies have reported that Tregs are downregulated in patients with ACS [11, 12], whereas others reported different results [13–15]. Furthermore, some authors have reported that the suppression function of CD4+CD25hi Tregs was not altered in patients [32], whereas others claim that Foxp3+ Tregs are not able to suppress the inflammatory cytokines [21, 22]. Whether CD25 and Foxp3, the once canonical typical markers of Tregs, truly are real specific markers for Tregs with a suppression function is now questioned. Given the controversy on the expression patterns and alteration trends of Tregs in ACS patients, as well as the significance of Tregs to ACS, we reanalyzed the expression of these markers. In line with some previous research [16, 17], no significant differences were found in CD4+CD25+ or CD4+CD25+Foxp3+ Tregs frequency among the three groups. However, the expression of Helios was remarkably decreased in both CD4+Foxp3+ and CD4+CD25+Foxp3+ T cells, and the estimated absolute number of CD4+Foxp3+Helios+ T cells was significantly decreased in ACS, too. Thus, it might be suggested that CD25 does not contribute to better distinguishing patients with ACS from others; examining Foxp3 coexpression with Helios rather than CD25 might be a better way to distinguish patients with ACS. Furthermore, our results show that better consistency in both protein level and mRNA level was found in Helios than in Foxp3, suggesting that Helios might be a more stable and reliable molecule for our research.

Helios was first suggested as a marker of nTregs by Sugimoto et al. in 2006 [38]. At that time, Helios was considered to be expressed exclusively on Foxp3+ Tregs. However, a few years later, it was shown that both Helios+ and Helios− cells exist in nTregs compartments [39, 40]. Helios was identified as a novel marker of activated nTregs with an important suppression function [36]. The role of Helios in CAD has not been explored yet, but given the immunosuppression defect characteristic of CAD and the immunosuppressive role of Helios, this molecule might contribute to the disease. Therefore, we conducted the current study to examine the expression of Helios in Tregs in patients with ACS. The decrease of Helios+ Tregs suggests that Helios might play a role in CAD. Moreover, the reduced TGF-beta1 level and the increased IL-6 level in plasma of patients with ACS are in line with previous studies [11, 12, 41], and their correlations with Helios+ Tregs were revealed in this study. Adding to the facts that the immunological suppression capacity of Tregs is partly dependent on TGF-beta1 [23] and that the proinflammatory cytokine IL-6 is a powerful independent risk factor for the development of CAD and ACS [42–44], it might be suggested that the reduction of Helios+ Tregs is associated with immunosuppressive deficiency in ACS, and Helios+ Tregs might play a role in CAD. A recent study also reported that IL-6 inhibited the TGF-beta–induced development of Helios+ Tregs [36]. With the low TGF-beta1 and Helios+ Tregs frequency and high IL-6 in our patients with ACS, it might be speculated that such a mechanism may participate in CAD progression. However, the truth is yet to be uncovered. Moreover, circulating HDL-C level positively correlated with Helios+ Treg. Given the anti-inflammatory and immunomodulatory properties of HDL-C [45] and its negative correlation with the frequency of proinflammatory T cell subsets [46], the correlation of Helios+ Tregs and HDL-C in our study suggests that Helios+ Tregs might play a role in immune modulation.

Further studies are still needed to define in detail the functional and biological importance of Helios+ T cells in patients with CAD and the potential use of the marker as a therapeutic target. However, because Foxp3 and Helios are intracellular proteins, it is impossible to sort living functional CD4+Foxp3+Helios+ cells in humans and conduct a suppression function assay to achieve further mechanism analysis.

## 5. Conclusions

Circulating Helios+ Tregs are significantly decreased in ACS, accompanied by increased IL-6 and decreased TGF-beta1 and HDL-C, which indicates that Helios+ Tregs may play a role in ACS. Examining Foxp3 coexpression with Helios rather than CD25 may be a better way to distinguish patients with ACS. Our results provide a new insight for Treg studies in ACS. Although this single study is not sufficient to prove that Helios+ Tregs play a protective role in ACS progression because we are not able to isolate Helios+ cells from patients due to the intracellular nature of Helios, we can nevertheless obtain evidence from in vitro research and animal experiments in subsequent work.

## Figures and Tables

**Table 1:** The calculation formula of estimated absolute number of different cell subsets.

Subsets	Calculation formula
CD4+Foxp3+	[total lymphocyte (cells/*μ*l)] × percentage (CD4+Foxp3+)
CD4+Foxp3+Helios+	[total lymphocyte (cells/*μ*l)] × percentage (CD4+Foxp3+) × percentage (Helios+/CD4+Foxp3+)
CD4+CD25+	[total lymphocyte (cells/*μ*l)] × percentage (CD4+CD25+)
CD4+CD25+Foxp3+	[total lymphocyte (cells/*μ*l)] × percentage (CD4+CD25+) × percentage (Foxp3+/CD4+CD25+)
CD4+CD25+Foxp3+Helios+	[total lymphocyte (cells/*μ*l)] × percentage (CD4+CD25+) × percentage (Foxp3+/CD4+CD25+) × percentage (Helios+/CD4+CD25+Foxp3+)

**Table 2:** Clinical data for the control, SA, and ACS patients.

	Control	SA	ACS	*P* value
	(*n* = 48)	(*n* = 30)	(*n* = 73)	
Gender (female/male)	(21/27)	(12/18)	(18/55)	0.068
Age (years)	54.73 ± 1.5	57.50 ± 1.5	58.32 ± 1.0	0.111
Risk factors, *n* (%)				
Current smoking	15 (31)	8 (26.7)	33 (45)	0.125
Diabetes mellitus	5 (10)	4 (13)	15 (21)	0.3
Hypertension	21 (44)	20 (67)	39 (53)	0.142
Hyperlipidemia	14 (29)	5 (17)	18 (27)	0.458
Statin	20 (42)	13 (43)	60 (82)	0.001
SBP (mmHg)	127.15 ± 2.44	129.03 ± 3.26	126.50 ± 2.30	0.821
DBP (mmHg)	80.29 ± 1.64	77.73 ± 1.90	76.97 ± 1.28	0.261
TC (mmol/l)	4.24 ± 0.14	3.97 ± 0.15	3.86 ± 0.11	0.082
HDL-C (mmol/l)	1.43 ± 0.06	1.38 ± 0.06	1.18 ± 0.03	0.001
LDL-C (mmol/l)	2.29 ± 0.11	2.06 ± 0.11	2.09 ± 0.8	0.203
TG (mmol/l)	1.48 ± 0.79	1.61 ± 1.24	1.41 ± 0.10	0.576
CK-MB (ng/ml)	0.5 ± 0.03	0.8 ± 0.05	98.5 ± 9.5	0.001
Troponin I (pg/ml)	3.80 ± 0.12	5.2 ± 0.18	36538.2 ± 582.1	0.001

The values are presented as mean ± SEM or number (%). SA: stable angina; ACS: acute coronary syndrome; SBP: systolic blood pressure; DBP: diastolic blood pressure; TC: total cholesterol; HDL-C: high-density lipoprotein cholesterol; LDL-C: low-density lipoprotein cholesterol; TG: triglyceride.

**Table 3:** The correlations of Helios+ Treg proportion and cytokines in each group.

	Control	SA	ACS
IL-6	*r* = −0.155 *p* = 0.293	*r* = −0.532 *p* = 0.002	*r* = −0.114 *p* = 0.338

TGF-beta1	*r* = 0.257 *p* = 0.077	*r* = 0.216 *p* = 0.052	*r* = 0.219 *p* = 0.063
